# Locoregional Treatments in Cholangiocarcinoma and Combined Hepatocellular Cholangiocarcinoma

**DOI:** 10.3390/cancers13133336

**Published:** 2021-07-02

**Authors:** Matteo Renzulli, Daryl Ramai, Jameel Singh, Samridhi Sinha, Nicolò Brandi, Anna Maria Ierardi, Elisa Albertini, Rodolfo Sacco, Antonio Facciorusso, Rita Golfieri

**Affiliations:** 1 Department of Radiology, IRCCS Azienda Ospedaliero-Universitaria di Bologna, Via Albertoni 15, 40138 Bologna, Italy; nicolo.brandi@studio.unibo.it (N.B.); rita.golfieri@unibo.it (R.G.); 2 Department of Internal Medicine, The Brooklyn Hospital Center, Brooklyn, New York, NY 11201, USA; dramai@tbh.org (D.R.); ssinha@tbh.org (S.S.); 3 Department of Internal Medicine, Mather Hospital, Northwell Health, Port Jefferson, New York, NY 11777, USA; jameel.k.singh@gmail.com; 4 Diagnostic and Interventional Radiology, ASST Santi Paolo e Carlo, San Paolo Hospital, 20142 Milan, Italy; amierardi@yahoo.it; 5 Pathology Unit, IRCCS Azienda Ospedaliero-Universitaria di Bologna, Via Albertoni 15, 40138 Bologna, Italy; elisa.albertini2@studio.unibo.it; 6 Section of Gastroenterology, Department of Medical Sciences, University of Foggia, 71122 Foggia, Italy; r.sacco@ao-pisa.toscana.it (R.S.); antonio.facciorusso@virgilio.it (A.F.)

**Keywords:** cholangiocarcinoma, combined hepatocellular cholangiocarcinoma, biliary tract

## Abstract

**Simple Summary:**

Cholangiocarcinoma is an aggressive primary cancer of the biliary tree. Combined hepatocellular cholangiocarcinoma is also a primary liver malignancy but displays properties both of cholangiocarcinoma and hepatocellular carcinoma. Liver resection is the mainstay treatment; however, some patients are not surgical candidates. Locoregional therapies have emerged with the goal of providing local cancer treatment and control. We review different locoregional strategies for treating cholangiocarcinoma and combined hepatocellular cholangiocarcinoma.

**Abstract:**

Cholangiocarcinoma (CCA) is a primary and aggressive cancer of the biliary tree. Combined hepatocellular cholangiocarcinoma (CHC) is a distinctive primary liver malignancy which has properties of both hepatocytic and cholangiocytic differentiation. CHC appears to have a worse prognosis compared to hepatocellular carcinoma, and similar to that of intrahepatic CCA. While significant advances have been made in understanding the pathophysiology and treatment of these two tumor types, their prognosis remains poor. Currently, liver resection is the primary treatment modality; however, only a minority of patients are eligible for surgery. However, the use of locoregional therapies proves an alternative approach to treating locally advanced disease with the aim of converting to resectability or even transplantation. Locoregional therapies such as transarterial chemoembolization (TACE), selective internal radiation therapy (SIRT), radiofrequency ablation (RFA), and photodynamic therapy (PDT) can provide patients with tumor control and increase the chances of survival. In this review, we appraise the evidence surrounding the use of locoregional therapies in treating patients with CCA and CHC.

## 1. Cholangiocarcinoma

**Figure 1:**
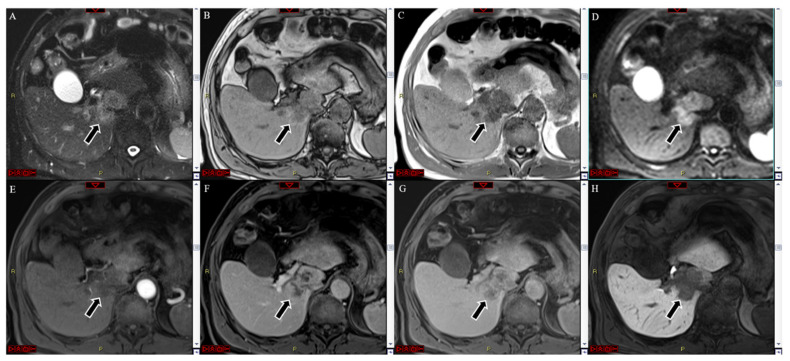
MRI with Gd-EOB-DTPA of a histologically confirmed intra-hepatic cholangiocarcinoma located in the segment I of the liver (arrow). The lesion showed hyperintensity at the T2-weighted image (**A**), whereas it was hypo both in in- and out-of-phase T1-weighted images (**B**,**C**), coupled with strong hyperintensity in the diffusion-weighted image (**D**). No significant hyperenhancement was seen in the arterial phase (**E**). A heterogeneous centripetal enhancement was detected during the portal and late venous phases (**F**,**G**), without any washout, but the center of the lesion was continuously hypointense. In the hepatobiliary phase, the lesion appeared hypointense (**H**).

Cholangiocarcinoma (CCA) is a biliary malignancy that accounts for 3% of gastrointestinal malignancies and 15% of primary liver cancers [1]. CCA can be subclassified based on its anatomical site of origin. These classifications include intrahepatic CCA (iCCA) (Figure 1A–H), perihilar CCA (pCCA), and distal CCA (dCCA) [2]. The overall incidence of CCA has increased over recent decades, and the percentage of patients who survive 5 years after diagnosis remains unchanged (approximately 10%) [3,4,5].

A US Surveillance, Epidemiology, and End Results (SEER) study reported an overall incidence of CCA of 11.98 [95% CI, 11.79–12.16] per 1,000,000 over the period from 2000 to 2015 [6]. The highest incidence rates were found among males (13.94 [95% CI, 13.64–14.25]), patients older than 65 years (63.43 [95% CI, 62.21–64.68]), and Asians (17.78 [95% CI, 17.00–18.58]) [6]. The overall mortality was shown to be 10.30 [95% CI, 10.118–10.47]. Furthermore, this report suggested that the highest risk of mortality was among males (hazard ratio (HR) 12.16 [95% CI 11.866–12.460]), patients older than 65 years (HR 57.85 [95% CI, 56.65–59.08]), and Asians (HR 14.96 [95% CI, 14.25–15.71]).

Globally, the incidence of CCA remains highest in northeast Thailand [7,8,9]. The age-standardized incidence rates (ASIR) suggest approximately 100 per 100,000 individuals for men and 50 per 100,000 for women [7,8,9]. In the West, the ASIR is 0.5–2.0 per 100,000 individuals [9,10]. These higher rates can be attributed to Opisthorchis viverrini infection, an endemic liver fluke [10].

The prognosis remains poor despite the advances made in recent decades to understand these complex malignancies and new treatment strategies. Liver resection (LR) is the definitive treatment, but only a few patients are candidates for surgery. For locally advanced CCA in patients who are not LR candidates, neoadjuvant therapies can be used to reduce the tumor burden and allow these patients to eventually be resection or transplantation candidates. These neoadjuvant therapies include transarterial chemoembolization (TACE), selective internal radiation therapy (SIRT), radiofrequency ablation (RFA), and photodynamic therapy (PDT), which can control local tumors and avoid systemic treatment side effects.

### 1.1. Pathogenesis of Cholangiocarcinoma

Chronic inflammation and cholestasis are the most common causes for developing cholangiocarcinoma. Increased exposure of cholangiocytes to inflammatory markers such as IL-6, tumor necrosis factor-a, cyclo-oxygenase-2, and Wnt can lead to mutations of tumor suppressor genes and proto-oncogenes and to DNA mismatch repairs. Accumulation of bile acids and the reduced pH can also lead to increased apoptosis via ERK1/2, Akt, and NF-KB pathways that increase cell proliferation.

Cholangiocarcinomas are anatomically classified; however, the cells of origin (cholangiocytes, peribiliary glands, hepatic progenitor cells or hepatocytes) allow for different forms of classifications which may allow for better prediction of tumor behavior [11]. Worldwide, the incidence of iCCA is rising, while the incidence of perihilar or distal CC is decreasing [12]. Incidence rates also vary significantly in different countries, with Switzerland having an incidence rate of 0.45 per 100,000, while Italy has an incidence rate of 3.36 per 100,000 [13]. However, the highest incidence rates occur in Asia due to the prevalence of parasitic liver infections (approximately 85 per 100,000 in northeast Thailand) [11]. 

Intraductal papillary neoplasms localized to the bile duct show a stepwise progression of increasing dysplasia which supports the adenoma–dysplasia–carcinoma sequence. Biliary intraepithelial neoplasia, which arises from flat lesions of the cholangiocytes and peribiliary glands of the bile duct, also supports this hypothesis [11]. 

Carcinogenesis of CCA can be attributed to transformed glucose metabolism. Cancer cells promote increased glucose uptake and subsequent glycolysis. When pyruvate is generated, cell proliferation is also promoted, which has been supported by findings of higher lactate dehydrogenase in CCA [14]. Among risk factors for CCA include cholestatic liver diseases which increase inflammation and, ultimately, lead to overexposure of cholangiocytes to bile acids, which leads to abnormal cell proliferation and cholangiocarcinogenesis [11]. Liver cirrhosis has also been shown to have an increased risk of intrahepatic cholangiocarcinoma, with an OR of 22.9 (95% CI 18.2–28.8). (18) It is hypothesized that this is due to chronic inflammation as well as increased cell turnover and progressive fibrosis. Cirrhosis is also associated with an increased risk of perihilar and extrahepatic CCA [15]. It is hypothesized that decreased levels of bile acid excretion found in cirrhosis can lead to gut microbiome dysbiosis and contribute to a pro-inflammatory state [16,17].

Biliary stone disease has also been shown to contribute to increased risks of both iCCA and eCCA. A SEER database analysis demonstrated an OR of 6.94 (95% CI 5.64–8.54) for ICC and 14.22 (95% CI of 12.48–16.20) for ECC [18]. The proposed hypothesis is that the carcinogenesis stems from impaired biliary drainage and recurrent bacterial infections. (18) Additionally, chronic infections such as hepatitis B and C as well as liver fluke infections in endemic areas also contribute to the increased risk of ICC which is attributed to sustained inflammation that either directly or indirectly affects the biliary tree and leads to mutagenesis and cancer development [11]. Metabolic disorders increase the risk of both iCCA and eCCA. Diabetes mellitus was shown to have an OR of 1.74 (95% CI of 1.62–1.87) [19]. Additionally, non-alcoholic fatty liver disease (NAFLD) has been shown to have a 3-fold increase for the risk of IC (OR 3.52 95% CI 2.87–4.32) and ECC (2.93, 95% CI of 2.42–3.55) [15].

### 1.2. Radiofrequency Ablation

In patients who carry a diagnosis of iCCA, surgical resection with histologically negative margins shows the best outcomes in regard to survival and is the preferred treatment [20,21]. Radiofrequency ablation (RFA) has been used when the tumor is unresectable or when recurrent tumors occur after resection of the primary tumor [22]. Previous studies have reported delivery of RFA percutaneously through ultrasound guidance as well as the use of open or laparoscopic RFA techniques for ablation of large tumors [22]. The efficacy of RFA is based on whether or not complete tumor ablation is achieved. Additionally, operator experience has been associated as a predictor of morbidity and complete ablation rate [22]. Of note, to achieve an optimal ablative field, a single electrode is normally used for smaller lesions (measuring up to 2–3 cm in diameter), and multiple or clustered electrodes are used for larger lesions (3–3.5 cm in diameter) and require ablative margins of 0.5–1 cm [23,24]. 

A 2015 meta-analysis revealed pooled survival rates of 82% (95% CI, 72–90%), 47% (95% CI, 28–65%), and 24% (95% CI, 11–40%) [24]. Han et al. also suggested that the overall hospital stay length, cost of treatment, and complication risk are less with RFA than with surgery [24]. The median survival time was reported to be 20–60 months (Table 1). Major complications include the possibility of liver abscess, biliary strictures, or bleeding. Minor complications included post-ablative syndrome and were controlled with conservative management [24]. 

### 1.3. Photodynamic Therapy

Photodynamic therapy (PDT) is a tumor-specific ablation therapy that is the standard of care for nonresectable cholangiocarcinoma [25]. The mechanism of action involves treatment with a photosensitive drug with affinity for the target tissue and irritation with light of a specific wavelength that causes death of tumor cells via production of oxygen free radicals. (6) Unresectable cholangiocarcinoma has a median survival time of 3 months with no intervention and 4–10 months with biliary drainage [25,26]. A recent meta-analysis compared the survival benefit and quality of life between patients who received PDT with biliary stenting vs. patients who received only biliary stenting (BS) [25]. The overall survival for patients who received PDT and BS vs. patients who received BS only was 413.04 days (95% CI: 349.54–476.54) and 183.41 days (95% CI: 136.81–230.02), respectively [25]. Moole et al.’s study also supported a high rate of successful biliary drainage in patients who had received PDT vs. those who received BS only, with an OR of 4.39 (95% CI: 2.35–8.19) (Table 1) [25]. Overall, PDT is well tolerated with a minimal side effect profile, but patients do stand the risk of cholangitis according to Moole’s 2017 meta-analysis. It should be noted that all patients who received PDT also received biliary stenting, and further studies would be needed to clearly identify the risk of cholangitis with PDT.

### 1.4. Transarterial Chemoembolization

Transarterial chemoembolization (TACE) has also been studied as a neoadjuvant approach in cholangiocarcinoma patients. TACE is an intra-arterial modality used in unresectable ICC that utilizes chemotherapeutics and an oil-based contrast agent in the tumor-supplying branch of the hepatic artery and subsequent embolizing agent [27]. A 2007 study by Herber et al. showed no change in disease in 60% of patients and a partial response in 7% of patients, with a median overall survival (OS) of 21.1 months; however, this study had a small sample size consisting of 15 patients in total [28]. Vogl’s 2013 study suggested stable disease in 57% of 115 iCCA patients receiving TACE, with a median OS of 13 months [29] (Table 1). There have not been any recent trials conducted to evaluate the efficacy of TACE in patients with iCCA [21].

### 1.5. Selective Internal Radiotherapy

Radioembolization via yttrium-90, referred to as selective internal radiotherapy (SIRT), provides locoregional treatment for primary tumors and metastatic disease [10,30]. Prior studies have suggested a median response rate which ranged from 5% to 36% and a median OS of 9 to 22 months, which has been attributed to the heterogeneity of the study population [30]. Edeline et al. published their findings of a phase 2 clinical trial that compared radioembolization and chemotherapy as first-line treatment for locally advanced iCCA [30]. Their study included 45 patients and suggested the disease control rate at 3 months was 98% (95% CI, 89–99%), and the median progression-free survival (PFS) was 14 months (95% CI, 8–17 months), with a 12-month PFS of 55% (95% CI, 40–71%). Additionally, median OS was 22 months (95% CI, 14–52 months), with a 12-month OS rate of 75% (95% CI, 62–89%) and 24-month OS rate of 45% (95% CI, 30–61%) (Table 1) [30]. TARE is an important modality with an overall good OS and low adverse events. However, future trials are needed to compare and assess its efficacy with other modalities [31].

### 1.6. Microwave Ablation

Microwave ablation (MWA) evolved as an alternative to RFA that allows for larger ablation zones and can be used in tissues in which RFA would not be successful, such as charred desiccated tissue [32,33]. The largest retrospective study consisting of 107 patients with primary or recurrent iCCA who underwent MWA found an OS at 1, 3, and 5 years of 93.5%, 39.6%, and 7.9%, respectively [34]. Additionally, Yu et al. reported a 60% survival at 1 and 2 years in 15 patients following MWA for iCCA (Table 1) [35]. A retrospective study found that MWA combined with transarterial chemoembolization in 26 patients showed a 6-, 12-, and 24-month survival rate of 88.5%, 69.2%, and 61.5%, respectively, with no major complications reported [36]. A 2019 retrospective study which included a total of 121 patients who underwent US-guided percutaneous MWA found that the 5-year OS rates were 23.7% after MWA and 21.8% after surgical resection (SR), and also that the complication rates were higher in patients who underwent SR (SR, 13.8% vs. MWA 5.3%, *p* < 0.001) [37]. These studies suggest that ablative therapies may prolong survival in patients with iCCA, but further investigation is warranted [38].

**Table 1:** Summary of studies on the use of locoregional therapy for cholangiocarcinoma.

Reference	Patients	Treatment	Responders	Median PFS (Months)	Median OS (Months)	Tumor Progression (%)
Han et al. (2015) [24]	84	Radiofrequency Ablation	-	-	1-year survival rate—82% (95% CI 72–90%) 3-year survival rate—47% (95% CI 28–65%) 5-year survival rate—24% (95% CI 11–40%	21% (CI 95% 13–30%)
Moole et al. (2017) [25]	297	Photodynamic Therapy	-	-	13.6 months (95% CI 11.47–15.67)	-
Herber et al. (2007) [28]	15	TACE	-	-	21.1 months (95% CI 9.4–32.5 months)	4/15 patients—8.2%
Vogl et al. (2012) [29]	115	TACE	Partial Response 8.7%	7 months	13 months	-
Edeline et al. (2019) [30]	41	Selective Internal Radiotherapy	41% (95% CI 28–55%)	14 months (95% CI 8–17 months)	22 months (95% CI 14–52 months)	-
Zhang et al. (2016) [34]	107	MWA	-	8.9 months (95% CI 6.5–11.3 months)	28.0 months (95% CI 23.7–32.2 months)	-
Yu et al. (2011) [35]	15	MWA	-	-	10 months	25% (6/24 nodules total in 15 patients)
Ohkawa et al. (2014) [39]	20	Proton Beam Therapy	-	-	27.5 months in curative group 9.6 months in palliative group	-
Hong et al. (2016) [40]	37	Proton Beam Therapy	-	8.4 months (95% CI 5–15.7 months)	22.5 (95% CI 12.4–49.7 months)	-
Frankulli et al. (2019) [41]	182	Stereotactic Body Radiation	-	-	1-year survival rate of 57.1% (95% CI 45–58%)	-
Queen et al. (2014) [42]	106	Endoscopy	-	-	2.89 months (95% CI 0.09–30 months)	-

### 1.7. Irreversible Electroporation

Irreversible electroporation (IRE) is a newer form of ablative technology that uses a high electrical voltage rather than thermal-based ablation such as RFA, MWA, and cryoablation [33]. There is a lack of data for IRE due to the small amount of unresectable primary or recurrent iCCAs that meet criteria for IRE treatment. A systematic review of 9 studies including 21 patients with iCCA treated with IRE found a reduction in tumor size; however, the subtype of cholangiocarcinoma was not mentioned in the study [43]. IRE provides nonthermal technology which may be beneficial in primary or recurrent iCCA when there is proximity to sensitive structures [33]. 

### 1.8. Proton Beam Therapy

Proton beam therapy (PBT) is increasingly used, has been shown to yield excellent dose localization to target tissues, and avoids irradiation of surrounding organs [44]. Ohkawa et al. reported 20 patients diagnosed with iCCA. A total of 12 of these patients were treated for cure and 8 for palliation, and they found median survival rates of 27.5 months and 9.6 months, respectively [39]. A phase 2 clinical trial reported a median OS of 23 months and a median PFS of 10 months (Table 1) [40]. Despite the results of previous studies using PBT, further clinical trials are warranted to further evaluate the use of PBT in iCCA. 

### 1.9. Stereotactic Body Radiotherapy

With the evolution of external beam radiotherapy, use of stereotactic body radiotherapy (SRBT) has been proposed for GI tumors. A 2019 systematic review consisting of nine studies suggested a 1-year pooled OS of 58.3% (CI 95% 50.2–66.1%), and the 1-year OS was 57.1% (CI 95% 45.0–58.0%) for iCCA (Table 1) [41]. Among the severe complications, cholangitis, abnormal LFTs, and duodenal obstruction, as well as transient biliary obstruction, were reported [45,46,47]. Due to the lack of data, further studies would be warranted to better define the role of SBRT in iCCA.

### 1.10. Endoscopy

Patients who are not surgical candidates may benefit from palliative endoscopic stent therapy. The median survival of patients without biliary stent placement has been shown to be lower than that for those with biliary drainage [47]. Additionally, endoscopic biliary drainage improves the quality of life by relieving jaundice and associated symptoms of jaundice such as diarrhea, sleep disturbance, anorexia, and pruritis [48]. Among the complications of stent therapy includes clogging with stones or obstruction with tumor ingrowth or overgrowth [48]. Despite complications, stenting can improve the quality of life in patients with cholangiocarcinoma (Table 1) [42].

## 2. Combined Hepatocellular Cholangiocarcinoma

**Figure 2:**
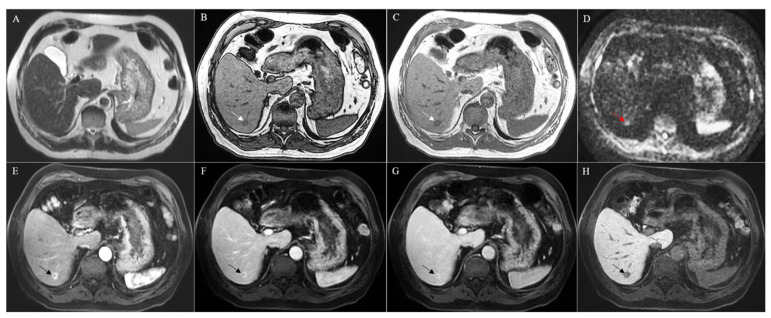
MRI with Gd-EOB-DTPA of a histologically confirmed combined hepatocellular cholangiocarcinoma located in the segment VI of the liver (arrow). The lesion presented without strong hyperintensity at the T2-weighted image (**A**) but showed hypointensity both in in- and out-of-phase T1-weighted images (**B**,**C**) and strong hyperintensity in the diffusion-weighted image (**D**). A strong hyperenhancement was evident during the arterial phase (**E**), followed by a persistent enhancement during the portal and late venous phases (**F**,**G**). In the hepatobiliary phase, the lesion appeared hypointense (**H**).

**Figure 3:**
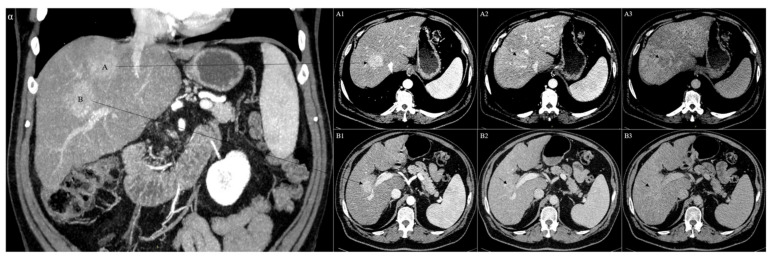
CT of two synchronous and separate epicenters of histologically confirmed hepatocellular carcinoma and combined hepatocellular cholangiocarcinoma in the same liver (**A**,**B**). In particular, the hepatocellular carcinoma was located in the segment VIII of the liver and presented an arterial phase hyperenhancement (**A1**), followed by an initial wash-out during the portal phase (**A2**), which became more evident during the late delayed phase (**A3**). On the contrary, the combined hepatocellular cholangiocarcinoma was located in the segment VI of the liver and showed a strong hyperenhancement during the arterial phase (**B1**), followed by a persistent enhancement during the portal phase (**B2**) and no wash-out during the late delayed phase (**B3**).

Combined or mixed hepatocellular cholangiocarcinoma (CHC) is a class of liver cancer that shares characteristics of HCC and CC [49,50,51]. It accounts for an incidence of 0.4–14.2% of primary liver carcinomas (PLC) [52,53,54,55,56]. The earliest pathological classifications provided in 1949 by Allen and Lisa consisted of three subtypes: type A, characterized by synchronous but separate and individual epicenters of both HCC and CC in one liver; type B, composed of mixed distinguished foci of HCC and CC; and type C, consisting of both HCC and CC that stem from the same tumor (Figure 2 A–H) [55]. Nonetheless, CHC and HCC can also coexist in the same liver simultaneously (Figure 3A–B). Recently, the WHO classification reported CHC as a distinct entity and identified two main subtypes: the classical type, and CHC with stem cell features [51]. 

Mutations of TP53, TERT promoter, and ARID1A are common genetic anomalies in CHC. PTMS-AP1G1 is a fusion gene unique to CHC. Definitive diagnosis of CHC requires biopsy. Surgical resection is the treatment of choice, while liver transplantation for early-stage CHC is shown to have favorable outcomes. Surgery has been effective in both younger as well as older patients, and resection margins of >10 mm have been associated with longer disease-free survival in patients [55]. However, CHC remains an aggressive tumor with a poor prognosis despite the surgical approach and has a high rate of recurrence as well as a 5-year survival rate of 30% [55]. 

Therapy with gemcitabine shows favorable outcomes for advanced CHC, with a combined progression-free survival of 8.0 months [55,57]. Sorafenib monotherapy had a progression-free survival of only 4.8 months [55]. Locoregional treatments have been considered in CHC that is surgically unresectable with no clear guidelines. Inoperability is usually defined as large or multifocal tumors [58]. CHC follows a pathological course as with HCC. Portal and hepatic venous infiltration is observed in both CHC and HCC. However, CHC and CC share a similar metastatic pattern [50,59]. CHC is both an uncommon and aggressive form of PLC. There are no guidelines to suggest optimal management for CHC. Localized tumors are often managed with radical resection which has become the preferred treatment option, and advanced tumors are often treated with systemic therapies that overlap with treatment for iCCA and HCC [60].

In patients who have recurrent or inoperable CHC, it is nonsurgically treated with TACE, RFA, SIRT, and systemic chemotherapy. The data to suggest the benefit of loco-regional therapies are limited but have shown partial response rates and can allow for surgical resection and a possible survival benefit [61,62,63,64,65].

### 2.1. Pathogenesis of Combined Hepatocellular Cholangiocarcinoma

CHCs are a rare class of PLCs and can consist of clinicopathological and radiological features of both HCC and CC in the same tumor [66]. The primary hypothesis is that CHCs originate from bipotent hepatic progenitor cells (HPCs) with both hepatocellular and cholangiocellular differentiation [66]. An alternate hypothesis suggested is that there is redifferentiation or dedifferentiation of HCC to a biliary phenotype and vice versa, but this remains a controversial theory [67,68].

The prevalence of CHC varies and ranges from 0.4 to 14.2%, with increasing incidence in the last several years [69,70]. Spolverato et al. reported a doubling of the number of diagnosed CHC cases from 2004 to 2015, which was attributed to an increase in accurate diagnosis [71]. A SEER database analysis of 20,000 patients with primary liver cancer found the CHC had an incidence of 1.3% of total cases [72]. Additional retrospective population-based studies suggest an average age of diagnosis of 62.5 years and an overall incidence of 0.05 per 100,000 per year [73]. Western studies seemed to suggest a correlation between male sex and older age, which contradicted Eastern studies which showed a correlation with male sex but had an earlier age of diagnosis [65].

Histological classification of CHC has been studied. CHC has been recognized by the 2010 World Health Organization (WHO) criteria as its own entity and subsequently divided into two different subtypes. The first of these subtypes is the most common. It is characterized by transition zones—a mix of HCC and CC. The second type contains stem cell features and consists of three different variants: (1) typical, with mature hepatocytes surrounded by HPC-like cells, (2) intermediate, with expression of both HCC and CC markers, and (3) cholangiolocellular (CLC), which consists of cells viewed as HPCs but organized into tubular cholangioles with desmoplastic stroma [67,74,75]. However, as of 2019, the WHO classification reclassified the CLC subtype to a subtype of small duct intrahepatic CC [76]. Molecular findings of CHC have not been clearly defined and carry a wide range of mutations which overlap between HCC and CC. However, whether CHC more closely resembles HCC or CC is a controversial and highly debated topic. Cazals-Hatem et al. demonstrated both LOH and TP53 mutations in more than 50% of CHC and CC cases, thus aligning CHC closer to CC [70]. Conversely, Joseph et al. suggested that CHC more closely resembles HCC [77]. While there are no typical CC alterations, TERT promoter mutations suggest an early component in CHC evolution as they were consistently observed both in HCC and CC components [68].

**Figure 4:**
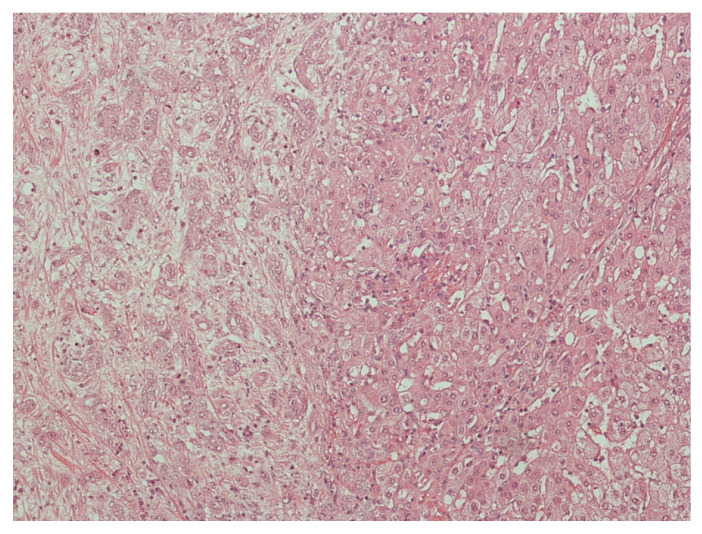
Histological features of combined hepatocellular cholangiocarcinoma (hematoxylin and eosin staining). Passage area between cholangiocarcinoma features (**left**) and HCC component (**right**).

Histologically, CHC commonly appears as a classical type which is characterized by variable merging of HCC and CC areas with areas of interface of unequivocal components (Figure 4) [78]. The HCC component is distinguished by the appearance of bile-producing cells (and canaliculi) with a granular eosinophilic cytoplasm and often contains intracytoplasmic fibrinogen, Mallory–Denk bodies, alpha1 antitrypsin, and fat globules [1]. Common immunohistochemical markers include HepPar1, glypican-3, CAM5.2, canalicular pCEA, and CD10. Typical stains for the biliary component include mucin/mucicarmine, pCEA, cytoplasmic CD10, AE1, MOC31, CK7, and CK19, the last two previously correlated with worse outcomes of HCC after liver transplantation [1,79]. Transition zones stain positively with CK7/CK19 and HepPar1 [1].

Risk factors for HCC and CC are similar to CHC, which include viral hepatitis, cirrhosis, and alcohol consumption. It is suggested that diabetes mellitus and obesity are possible confounders rather than risk factors for CHC as they contribute to non-alcoholic steatohepatitis and subsequent cirrhosis [80].

### 2.2. Therapy

Ablative therapies are minimally invasive and destroy normal tissue that arises with different conditions. This can be performed by using chemical, thermal, and other techniques. Chemical ablation utilizes ethanol or 5% acetic acid which induces local ischemia and necrosis. It is low cost but has high recurrence rates which can limit its use for lesions smaller in size. Chemical ablation is mainly used for HCC and can also be applied to neuroendocrine tumors. Thermal ablation is performed by applying cryotherapy or heat with RFA, PDT, MWA, or lasers [81]. 

Surgical resection remains the only curative option for patients with CHC and is dictated by multiple factors including the patient’s overall condition, tumor invasion, and the tumor’s anatomical location. Despite the aim of liver resection, CHC has been shown to have high recurrence rates and 5-year survival rates of 30% [62]. Studies have shown worse outcomes for CHC patients after resection than HCC, which can be attributed to the difference in biological features [62]. Liver transplantation has also been explored as a potential therapy and has been shown to have less favorable outcomes in CHC patients rather than in patients with HCC (survival rate 41.1% vs. 67%, *p* < 0.001) [62]. However, the use of locoregional therapy to reduce the local tumor burden may provide patients with benefits prior to liver resection or transplantation.

### 2.3. Chemoembolization

TACE, percutaneous ethanol injections (PEI), and RFA are applied in cases of unresectable HCC and recurrence following surgical resection [82,83]. Conversely, CHC tends to be less vascular and more fibrotic. As a result of these features, CHC is less responsive to TACE or PEI. However, RFA or cryoablation may be beneficial in select cases (Table 2) [63].

There are limited data on the efficacy of liver-directed therapies. A small retrospective study showed that 18 patients who received liver-directed therapies out of a cohort of 79 patients [83] were more likely to have increased tumor size compared to those undergoing surgical resection (8.9 vs. 5.8 cm) and a higher incidence of metastatic disease (33% vs. 8%) [83]. However, liver-directed therapy yielded an overall partial response rate of 47% (50% with radioembolization, 20% with TACE, and 66% with hepatic arterial infusional chemotherapy). The median progression-free survival and OS rates for the study cohort were 8.3 and 16.0 months, respectively. While the study was limited by the sample and retrospective design, it shows that liver-directed locoregional therapy may provide some patients with therapeutic benefits [83]. Furthermore, patients with down-sized tumors may be converted to surgical candidates. Kim et al. suggested that TACE produced a 12.3-month median overall survival and a tumor progression of 30% [84], while Na et al. suggested that TACE showed a median OS of 32.6 months and a median progression-free survival of 3.4 months (Table 2) [85].

In the future, as already demonstrated for HCC, the possible introduction of a subclassification of CHC stages could help in predicting the prognosis of patients and in choosing the more effective treatment option [86]. Techniques such as RFA or cryoablation might be of value in patients who have recurrence [87]. Overall, while the current data are very limited, the evidence appears to hint towards a therapeutic regimen which combines locoregional therapy such as RFA and platinum-containing drugs for nonresectable CHC. Systemic therapy has not been shown to have clear outcomes, with first-line treatments including sorafenib having a poorer OS when compared to sorafenib in combination with platinum-containing regimens (HR 15.83, 95% CI 2.25–111.43, *p* = 0.006) [62]. A small subgroup analysis revealed that patients treated with systemic chemotherapy compared to sorafenib and prior TACE or RFA tend to have better ECOG performance scores (63.9% vs. 24.3%, *p* < 0.001) [88]. Locoregional therapies would work to treat the HCC component of CHC, while platinum-based systemic chemotherapy would work against the CCA component of CHC. However, prospective clinical studies are needed to validate this theoretical regimen. Furthermore, therapies should include the integration of translational research, understand the genomic landscape of the tumor, and use molecular targeted treatments [62].

**Table 2:** Summary of studies on the use of locoregional therapy for combined hepatocellular cholangiocarcinoma.

Reference	Patients	Treatment	Responders	Median PFS (Months)	Median OS (Months)	Tumor Progression (%)
Fowler et al. (2015) [63]	79	TACE (6)	-	-	-	2 (30%)
		TARE (6)	47%	16	8.3	3 (50%)
		HAI pump (6)	-	-	-	0
		Systemic chemotherapy (28)	-	-	-	15 (44%)
		Surgery (33)	35 (70%)	16	12.3	0
Kim et al. (2010) [84]	50	TACE	35 (70%)	-	12.3	15(30%)
Chan et al. (2017) [89]	10	TARE	60% partial response and 40% stable disease	-	10.2 from 1st RE treatment and 17.7 from initial diagnosis	-
Na et al. (2018) [85]	42	TACE	Globally enhancing cHCC-CC—36% Peripherally enhancing cHCC-CC—0% HCC—35.6%	Globally enhancing cHCC-CC—4.7 Peripherally enhancing cHCC-CC—2.1 HCC—9.7	Globally enhancing cHCC-CC—52.8 Peripherally enhancing cHCC-CC—12.4 HCC—67.5	cHCC-CC—37(88.1%)

### 2.4. Transarterial Radioembolization

**Figure 5:**
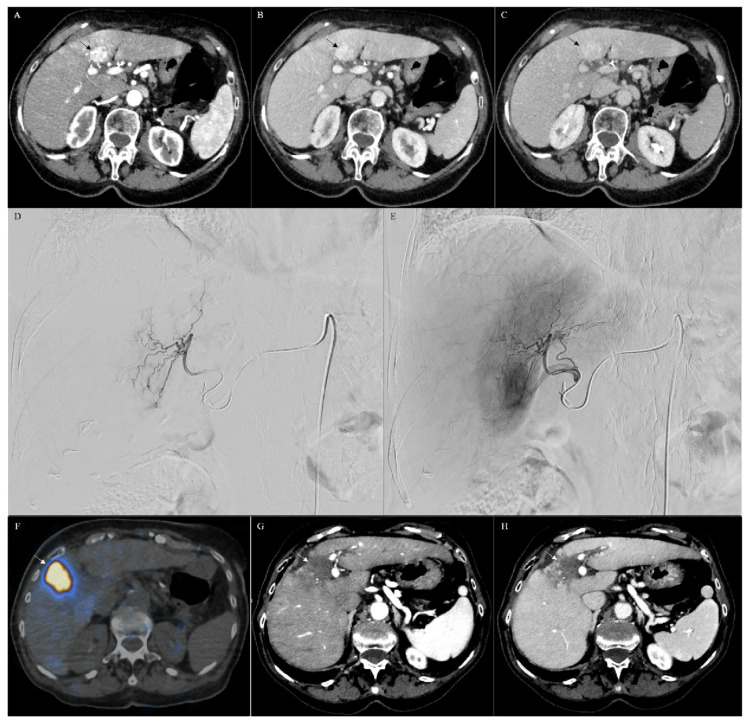
CT of a combined hepatocellular cholangiocarcinoma (arrow) (**A**–**C**). In particular, CT showed a lesion in the segment IV of the liver with a strong hyperenhancement during the arterial phase (**A**), followed by a persistent enhancement during the portal (**B**) and delayed phases (**C**), consistent with the imaging diagnosis of combined hepatocellular cholangiocarcinoma, thereafter confirmed with biopsy. The lesion was treated by using transarterial radioembolization (TARE). In particular, during the angiographic study (**D**,**E**), the artery feeding the liver segment IV was selectively catheterized (**D**), the lesion was confirmed with selective angiography (**E**), and the intra-arterial treatment was selectively performed. Positron emission/computerized tomography (PET/CT (**F**)) demonstrated that the treatment was correctly performed, covering all the lesion (arrow in (**F**)). CT scan, performed 6 months after TARE, demonstrated complete response of the lesion characterized by no vascularized area in the IV segment of the liver in the arterial (arrow in (**G**)) and in the venosus (arrow in (**H**)) phases.

Locoregional therapy can also be applied for palliative reasons with or without chemotherapy to provide symptomatic control [59,89,90]. Chan et al. reported a small study of 10 patients (median age 59 years; 6 men, 4 women) with histologically confirmed unresectable CHC treated with radioembolization using resin (6 patients) or glass (4 patients) microspheres [91]. From initial diagnosis and from the first radioembolization treatment, the median overall survivals were 10.2 and 17.7 months, respectively (Table 2). Tumor biomarker levels before and after showed a 60% median reduction (range 13–80%). According to RECIST, a partial response was observed in 60%, while 40% had a stable disease, compared to 100% using RECIST V1.1 [92]. The study shows that radioembolization may be a promising method for treating patients who are not initial candidates for resection (Figure 5A–H). Furthermore, radioembolization may be an option for patients with metastatic disease, though with a lower response rate [91,92].

## 3. Conclusions

Locoregional therapies provide local cancer treatment and control. While advances in recent decades have developed strategic therapies, there is a paucity of large clinical trials, particularly with patients diagnosed with CHC. Current clinical trials are likely to impact clinical practice and offer patients an improved quality of life and survival.
